# Planning nature-based solutions: Principles, steps, and insights

**DOI:** 10.1007/s13280-020-01365-1

**Published:** 2020-10-14

**Authors:** Christian Albert, Mario Brillinger, Paulina Guerrero, Sarah Gottwald, Jennifer Henze, Stefan Schmidt, Edward Ott, Barbara Schröter

**Affiliations:** 1 Chair for Environmental Analysis and Planning in Metropolitan Regions, Institute of Geography, Ruhr University Bochum, Universitaetsstr. 150, 44780 Bochum, Germany; 2 Institute of Environmental Planning, Leibniz University Hannover, Herrenhaeuser Str. 2, 30419 Hannover, Germany; 3 Leibniz-Centre for Agricultural Landscape Research – ZALF, Working Group ‘Governance of Ecosystem Services’, Eberswalder Str. 84, 15374 Müncheberg, Germany

**Keywords:** Ecosystem services, Environmental planning, Framework, Lahn river, Landscape planning, Spatial planning

## Abstract

Nature-based solutions (NBS) find increasing attention as actions to address societal challenges through harnessing ecological processes, yet knowledge gaps exist regarding approaches to landscape planning with NBS. This paper aims to provide suggestions of how planning NBS can be conceptualized and applied in practice. We develop a framework for planning NBS by merging insights from literature and a case study in the Lahn river landscape, Germany. Our framework relates to three key criteria that define NBS, and consists of six steps of planning: *Co-define setting*, *Understand challenges*, *Create visions and scenarios*, *Assess potential impacts*, *Develop solution strategies*, and *Realize and monitor*. Its implementation is guided by five principles, namely *Place-specificity*, *Evidence base*, *Integration*, *Equity,* and *Transdisciplinarity*. Drawing on the empirical insights from the case study, we suggest suitable methods and a checklist of supportive procedures for applying the framework in practice. Taken together, our framework can facilitate planning NBS and provides further steps towards mainstreaming.

**Electronic supplementary material:**

The online version of this article (10.1007/s13280-020-01365-1) contains supplementary material, which is available to authorized users.

## Introduction

The concept of nature-based solutions (NBS) has become a key topic of contemporary research around options for more sustainable development of cities and rural areas. After its introduction by the European Commission (2015) and the International Union for Conservation of Nature, IUCN, (Cohen-Shacham et al. 2016), the concept has received immense interest in the scientific community, with 298 articles on the subject published in international peer-reviewed journals in the last three years alone (2017–2019, Scopus search on March 3, 2020). NBS are commonly understood as ‘actions which are inspired by, supported by or copied from nature’ (European Commission 2015, p. 5), although several authors reflect on the implications of this definition, relations to similar terms such as green infrastructure, and potential variations (e.g. Albert et al. 2017, 2019; Nesshöver et al. 2017). NBS have been proposed as key opportunities for adapting to climate change (e.g. Kabisch et al. 2016; Frantzeskaki et al. 2019), to attain the sustainable development goals (Faivre et al. 2017), and more generally to contribute to a better future for people and nature (Maes and Jacobs 2017; Seddon et al. 2020).

Landscape and urban planning have been identified as important instruments to enhance the consideration and uptake of NBS in efforts for navigating spatial development (e.g. Raymond et al. 2017; Frantzeskaki 2019). More specifically, Albert et al. (2019) highlighted the complementary contributions of landscape planning and governance research in identifying, designing and implementing NBS. However, knowledge gaps exist regarding concepts and methods of planning NBS in practice (Kabisch et al. 2016; Kumar et al. 2020; Mendez et al. 2020). The European Commission (2015) urges that design and implementation of NBS should be co-produced with multi-stakeholders and lessons learnt should be shared with others. Relevant insights for developing such knowledge can be found in general frameworks of landscape planning (e.g. Steinitz 1990; Ahern 1999; von Haaren et al. 2019) and recent studies that focused on how landscape planning could help in implementing NBS similar concepts, such as green and blue infrastructure (e.g. Hansen and Pauleit 2014; Meerow and Newell 2017).

The aim of this paper is to provide evidence-based suggestions of how planning NBS could be conceptualized and applied in practice. To achieve our aim, we (i) propose a framework of planning NBS and (ii) exemplify and reflect upon the application of the framework in an empirical case study.

## Methods

We developed a conceptual framework inspired by Hansen et al. (2017) on planning green infrastructure. The framework contains three key elements, which form three themes of planning NBS: (i) criteria of NBS and additional characteristics, (ii) essential planning steps, and (iii) planning principles. Criteria are requirements that actions need to fulfill to be considered NBS. Additional characteristics are attributes frequently associated with NBS, which may or may not be present in each NBS considered in planning. For example, the claim that NBS contribute to job creation (European Commission 2015) will probably hold for some but not all NBS in practice. Planning steps describe components of a cycle of planning with NBS. Planning principles are basic theorems that guide the implementation of the steps for planning NBS.

In a second step, we gradually refined our framework using concepts and findings of NBS literature. A scoping review (Peters et al. 2015) was conducted in Scopus for planning NBS (for details see BOX S1). Contents from reviewed literature were coded using a standard template according to the three themes of planning NBS. Relationships between the themes of the framework were then either recreated or generated based on the evidence from the reviewed literature. Findings of the scoping review were synthesized in a qualitative, narrative way (Snilstveit et al. 2012).

Finally, we deductively analyzed a case study carried out within this transdisciplinary research project to exemplify the framework. The case study should substantiate the planning steps of the framework with insights on application-oriented methods. We also reflected upon the degree of consideration of the planning principles and NBS criteria within those steps. All authors first reflected individually on the degree of consideration to collect a range of interpretations. Afterwards, these reflections were synthesized and critically discussed before summarizing the final findings. We applied several measures to enhance the validity of our reflection, including a thorough documentation of each workshop (audio recordings of plenary and group work, photographs, note taking, and observation protocol). The selection of the case study was based on the complex and multifaceted societal challenges of the Lahn River landscape that are currently addressed by a transdisciplinary development project (www.lila-livinglahn.de/en/start), the relevance to the topic of planning NBS, the availability and access to data and knowledge, and the opportunity to collaborate with practice partners. The Lahn River landscape offers various challenges to which NBS have proved to be suitable solutions. The challenges include the loss of former floodplain areas for settlement and infrastructure, intensification of agriculture, ecological deficits of the river according to the Water Framework Directive, and relatively high flood risk compared to the river’s tributaries (LiLa 2019). In this context, NBS actions such as upstream forest development have proven to enhance downstream flood protection (Barth and Döll 2016).

## Conceptual framework

**Fig. 1:**
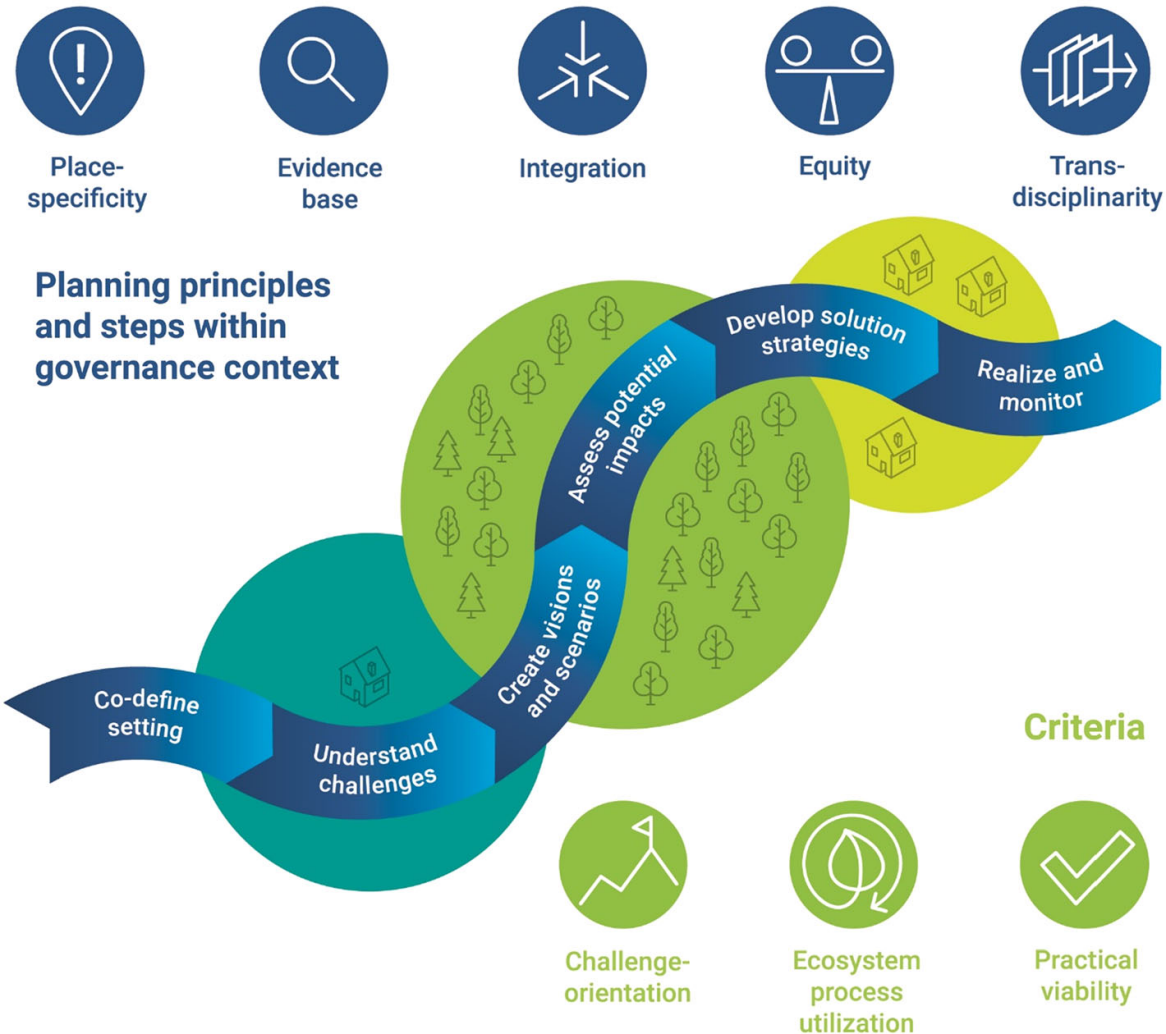
Conceptual framework of principles and steps for planning nature-based solutions (NBS) as well as key NBS criteria. Framework implementation needs to be specifically adapted to the respective biophysical and governance context. Iterative implementation, both between and across all planning steps, is crucial to ensure the incorporation of emerging knowledge

Our conceptual framework of planning NBS addresses three components (Fig. 1). First, we introduce criteria and common characteristics to define NBS as the substantive focus of the planning process. Second, we identify six steps that comprise a comprehensive approach to planning NBS, embedded in a given governance setting. Third, the procedural implementation of those steps is guided by five planning principles.

### Nature-based solutions criteria and characteristics

*Challenge-orientation* is the first of three criteria we understand as obligatory for NBS (Albert et al. 2019). It refers to an NBS’s contribution to alleviating a well-defined societal challenge which is frequently reflected in the literature. For example, NBS ‘address a variety of societal challenges in sustainable ways’ (European Commission 2015), ‘help meet various ethical, intellectual and relational challenges’ (Eggermont et al. 2015) and are ‘solutions for global challenges’ (Cohen-Shacham et al. 2016). Moreover, NBS have the potential to address diverse challenges such as biodiversity conservation (Connop et al. 2016), climate mitigation and adaptation (Wamsler et al. 2016), coastal protection and disaster risk reduction (Narayan et al. 2015), and urbanization (Connop et al. 2016; van der Jagt et al. 2019b).

*Ecosystem process utilization*, the second criterion, implies the use of ecological actions and events that link organisms and their environment (Albert et al. 2019). As put forward by the European Commission (2015), NBS should be ‘inspired by, supported by, or copied from nature.’ The degree of human intervention involved may vary, ranging from artificial solutions, such as biomimicry, to hybrid solutions and the protection of existing ecosystems (Eggermont et al. 2015; Arkema et al. 2017). However, the acceptable level of human intervention within NBS (Nesshöver et al. 2017) needs to be decided on a case-specific basis. Increasing consensus exists that NBS must protect essential ecosystem processes and resources and at least avoid the deterioration of the current state of ecosystems and biodiversity (Lennon and Scott 2016).

The third criterion, *practical viability*, refers to the embeddedness of NBS within governance and business models for implementation. To be viable, NBS need to be considered an integral part of governance models (Gulsrud et al. 2018; Cohen-Shacham et al. 2019). Suitable governance models for NBS may include global or bilateral treaties (Narayan et al. 2015; Cohen-Shacham et al. 2019), incentives or regulatory mechanisms (Faivre et al. 2017; Xing et al. 2017) and community-based approaches (Gulsrud et al. 2018). A business case for NBS appears best made with business models that optimize benefits for humans and ecological systems while achieving project cost-effectiveness (Fink 2016).

Publications mention *common NBS characteristics* that we regard as facultative, in contrast to the obligatory criteria defined above. One example is co-benefit generation. It is argued that NBS should provide ‘environmental, social and economic co-benefits’ (Loiseau et al. 2016; Calliari et al. 2019; Song et al. 2019). More specifically, potential co-benefits could include social cohesion, health improvement, urban heat island mitigation, increase in biodiversity, sustainable water management and job creation (Faivre et al. 2017; Xing et al. 2017; Gulsrud et al. 2018). Consequently, NBS are perceived as cross-sectorial solutions (Wendling et al. 2018) that serve several purposes (Haase et al. 2017) and ‘emphasize multifunctionality’ (Clabby 2016; Fink 2016). We argue that NBS co-benefits should be aspired, but not considered defining criteria. This would narrow the NBS concept unnecessarily and inhibit its development as an accepted alternative. Cost-effectiveness is another often-claimed NBS characteristic (e.g. Short et al. 2019; van der Jagt et al. 2019b). Compared with technical solutions, NBS are proposed to be either equally effective (Santoro et al. 2019) or more cost-effective (Raymond et al. 2017; Young et al. 2019). We argue that cost-effectiveness cannot be simply stipulated but requires a case-specific analysis.

### Six steps of planning nature-based solutions

We propose six consecutive steps of planning NBS that together comprise an adaptive planning cycle (Kato and Ahern 2008; Ahern et al. 2014). Actual implementation of NBS planning is likely iterative and will often cover only some steps. We describe an ideal process, acknowledging that several implementation variations will be appropriate in practice.

The first step, *Co-define setting*, includes the project kick-off and clarifies the context, overarching societal challenges, aims and processes of the project. This step paves the way for the practical viability of the NBS. It is usually undertaken by the planning team in close collaboration with key decision-makers and stakeholders (cf. Raymond et al. 2017; Izydorczyk et al. 2019). Ideally, the planning team would receive a mandate to enhance the legitimacy of the planning process and outputs. Sufficient funding for the planning process needs to be secured. The team identifies influential and affected stakeholders and devises a strategy for systematic and fair involvement (cf. Clabby 2016), facilitated by an independent moderator. To ensure consideration, planners need to link informal with formal planning instruments. Finally, the planning team clarifies expectations and limitations of stakeholder involvement in plan- and decision-making early on.

The second step, *Understand challenges*, relates to the respective defining criteria of NBS. In this planning step, the specific societal challenges framing the project need to be assessed in terms of existing problems or opportunities across spatial and temporal levels (cf. Raymond et al. 2017). A multi-dimensional assessment of the issues at stake is of particular importance. Societal dimensions include actors, networks, and problem perceptions. Legislative dimensions refer to existing aims, discrepancies between aims across institutions and hierarchical levels, and needs for institutional changes. Ecological dimensions involve risks of abrupt and irreversible ecosystem change. Furthermore, the dimension of human–nature-relationships needs to be considered, e.g. through ecosystem service delivery and demand or through sense of place. Understanding societal challenges such as water management or public health and well-being (Raymond et al. 2017) can be supported with systemic mapping. Tools such as causal loop diagramming or fuzzy cognitive mapping can identify stakeholders’ individual preferences and priorities for management (Pagano et al. 2019).

*Create visions and scenarios* comprises the identification and spatial localization of options for siting NBS within a given landscape context. Identifying appropriate solutions is the core aspect of the planning (Sarabi et al. 2019). It can begin with a joint definition of aims for landscape development in the future, based on challenges and problems previously identified and related to localized sustainable development goals (SDGs). The visions describe preferred future situations of landscape configurations and use. Scenario development may support discussion around the diversity of options with and without NBS and their likely impacts on various endpoints (Santoro et al. 2019). NBS, such as wetlands for flood protection, is a recent approach and not yet a widely accepted alternative to traditional measures such as dikes (cf. Brillinger et al. 2020). To integrate the new NBS concept, scenario methods may usefully stimulate creative and imaginative thinking (Alcamo et al. 2006; Albert et al. 2012) to consider different perspectives, and thus enable the uptake of NBS.

*Assess potential impacts* concerns the multidimensional evaluation of potential costs and benefits of either existing or to-be-implemented NBS, as well as other alternatives (cf. Raymond et al. 2017). This evaluation should follow the principles of multidimensional valuation (Pascual et al. 2017) and at least consider the need to recognize and—as much as possible—apply social and ecological valuations of decision-alternatives. The planning team can deliberately choose from qualitative or quantitative evaluation methods (Raymond et al. 2017). For example, Ourloglou et al. (2020) apply stream flow modelling and Augusto et al. (2020) combine meteorological, urban energy balance and hedonic pricing models to assess NBS effects. Both benefits and costs need to be carefully assessed and considered (Gómez Martín et al. 2020). This step provides NBS evidence as demanded by science (Raymond et al. 2017; Frantzeskaki et al. 2019) and the European Commission (2015).

*Develop solution strategies* concerns the design of feasible governance and business models for implementing preferred NBS scenarios, including a fair weighing of the pros and cons of implementation alternatives. The solution strategies need to target the place-specific context, and address the multiple barriers of implementation such as inadequate financial resources and regulations, institutional fragmentation, uncertainty regarding the implementation and effectiveness, and limited land and time availability (Sarabi et al. 2019). Policy mixes can facilitate effective resource allocation for NBS implementation (cf. Nesshöver et al. 2017). As part of policy mixes, integration of NBS in regional and local planning is critical (Zwierzchowska et al. 2019; Albert et al. 2020). Resources such as power, finances, and adequate personnel need to be secured (Pagano et al. 2019; Young et al. 2019). For implementation of NBS at the landscape scale, the creation of new regulatory bodies (Gulsrud et al. 2018) and more distributed but coordinated and integrated governance structures (cf. Wamsler et al. 2016; Dorst et al. 2019) are advisable.

Finally, *Realize and monitor* includes the implementation of first NBS actions and the critical monitoring of their effects. Design that creates a plan or specification for the implementation and monitoring may thereby act as common ground that connects the cycles of scientific inquiry and landscape change in practice (Nassauer and Opdam 2008). Erixon Aalto et al. (2018) found that design, as both a process and object, can facilitate realization through co-producing knowledge in action-oriented ways, for example through iterative prototyping, matrix models and comprehensive narratives. Inter- and transdisciplinary cooperation and design experimentation (Moosavi et al. 2019) may enable a close link between model-based assessments of NBS design options, and their monitoring and evaluation in subsequent implementation. To showcase NBS effects and facilitate upscaling, projects should be prioritized that are representative of specific conditions in the case study area, relatively easy to implement with available funding, and capitalize on previous success and evidence. Systematic monitoring allows for learning and adaptive governance (Folke et al. 2005; Molenveld and van Buuren 2019).

### Five principles of planning nature-based solutions

Our framework suggests that the six steps of planning NBS should follow five key guiding principles that may enhance the likelihood of successful implementation: *Place-specificity*, *Evidence base*, *Integration*, *Equity*, and *Transdisciplinarity*.

*Place-specificity* is essential, as both societal challenges and potential NBS are always context specific. NBS tend to be bound to a specific place (Albert et al. 2019; Colléony and Shwartz 2019; Young et al. 2019) so that planning with NBS needs to adapt general solutions to local conditions and challenges to ensure resource efficiency and resilience to change (European Commission 2015; Narayan et al. 2015; Raymond et al. 2017; Dorst et al. 2019). Failing to consider local conditions may cause negative effects (Guerrero et al. 2018), and mismatches between a particular action and the socio-spatial context might imply that the envisaged NBS no longer qualifies as a ‘solution’ (Young et al. 2019). Vice-versa, establishing NBS also can help shape a new sense of place (Frantzeskaki 2019).

NBS planning needs to be *based on evidence*, i.e. available information and knowledge for a specific NBS in a particular setting in order to infer reliable recommendations and actions (Calliari et al. 2019). For evidence-based practice, skills are required to find reliable research evidence, to apply it to specific application cases and to evaluate the effects of empirically grounded interventions (Sackett et al. 2000). Different approaches for the consideration of evidence are mentioned in literature (e.g. Sutherland et al. 2004; Ferraro and Pattanayak 2006; Mupepele et al. 2015). However, empirical evidence on NBS’ multi-dimensional effectiveness and their multiple benefits and co-benefits is missing (Raymond et al. 2017; Pagano et al. 2019). There is substantial merit in conducting more evaluation and monitoring studies on NBS efficiency and effectiveness. When only limited ‘hard’ evidence is available, expert judgements are required (Higgs et al. 2001).

*Integration* means considering thematically related approaches (Cohen-Shacham et al. 2019), temporal, spatial and sectoral scales within the planning process and policies in the governance context. The design and planning of NBS can integrate insights and methods from various established ecosystem-based approaches such as ecosystem services, green and blue infrastructure, ecological engineering, ecosystem-based management and natural capital (see Nesshöver et al. 2017). It can also integrate assessments of social and economic benefits of solutions that combine technical, business, finance, governance, regulatory and social innovation (European Commission 2015; Raymond et al. 2017; Xing et al. 2017).

*Integration* across spatial scales is essential concerning which NBS can deliver social and ecological benefits and address societal challenges (Cohen-Shacham et al. 2019; Dorst et al. 2019). Multi-directional effects may occur across different scales (Arkema et al. 2017; Raymond et al. 2017). For instance, some NBS produce additional co-benefits when up-scaled and may contribute to broader and multiple policy goals (Geneletti and Zardo 2016; Raymond et al. 2017). Others might be effective in addressing small-scale, short-term societal challenges, but may not have the same effectiveness on larger-scales and over the long-term (Arkema et al. 2017; Raymond et al. 2017). They may even interfere with other policy goals (Haase et al. 2017; Raymond et al. 2017). Integrative multi-scale approaches avoid overlooking multi-directional effects of NBS and allow for effective NBS planning and implementation (Arkema et al. 2017). While being integrative in considering interactions across scales (Bridgewater 2018), planning with NBS should focus on the landscape-scale and consider interconnected networks of multiple habitats or (semi-)natural areas to function effectively (Loiseau et al. 2016; Arkema et al. 2017).

Integrative planning approaches should also account for temporal scales. Often, NBS effects may fluctuate over time (Calliari et al. 2019; Cohen-Shacham et al. 2019) and need longer time periods to be effective in delivering a full range of potential ecosystem services and societal benefits (Maes and Jacobs 2017; Cohen-Shacham et al. 2019) when compared with effects from technical, hard engineering solutions (Guerrero et al. 2018).

*Equity* can be understood along four interlinked dimensions: recognition, procedure, distribution and context. This means recognizing the rights, values and interests of different actors, building on inclusive and effective participation of all relevant actors, equal distribution of costs and benefits amongst the actors, and taking into account the action-shaping context created by the pre-existing political, economic, and social conditions (Schreckenberg et al. 2016; Friedman et al. 2018). We propose to emphasize *Equity* as a principle regarding both organized participation and the planning outputs that are being delivered. The planning team should aim at organizing a socially inclusive planning process (Song et al. 2019; van der Jagt et al. 2019a) and promote transparency and broad participation in a fair and equitable way (Cohen-Shacham et al. 2019). The planning outputs need to consider aspects of environmental justice (van der Jagt et al. 2019b), so that NBS can lead to a greener and more sustainable society (Gómez Martín et al. 2020).

*Transdisciplinarity* refers to the cooperation of researchers from different disciplines and non-academic participants to create new knowledge and answer a common question (Tress et al. 2005). In the context of planning with NBS, it may be understood as the systematic involvement of diverse knowledge holders in the co-design and implementation of the planning process. As such, it has been widely identified as one of the key success factors of planning and implementing NBS (e.g. Nesshöver et al. 2017; Raymond et al. 2017; Calliari et al. 2019). The transdisciplinary planning process needs to apply diverse collaborative planning approaches to community engagement and citizen empowerment (Wamsler et al. 2016; Faivre et al. 2017). Numerous authors propose applying specific measures for promoting knowledge co-production and co-creation processes (Frantzeskaki et al. 2019; Short et al. 2019). *Transdisciplinarity* could be facilitated by the function of NBS as a boundary object that is robust but flexible enough to allow different stakeholders to develop a common language for cooperation (Dorst et al. 2019). However, ‘relabelling’ related concepts and misusing the NBS concept have to be prevented to avoid misunderstanding, duplication and unintended consequences (Nesshöver et al. 2017). A common vocabulary needs to be developed to effectively share and co-generate information on NBS. Additionally, participatory techniques should be used to raise awareness and motivation (Pagano et al. 2019). Systematic involvement does not imply that all planning activities need to be conducted by all people involved. Instead, phases of disciplinary, interdisciplinary and transdisciplinary collaboration should be strategically interwoven over the planning process to make the best use of complementary contributions.

## Reflection of a case study in practice

### The PlanSmart project case study

**Fig. 2:**
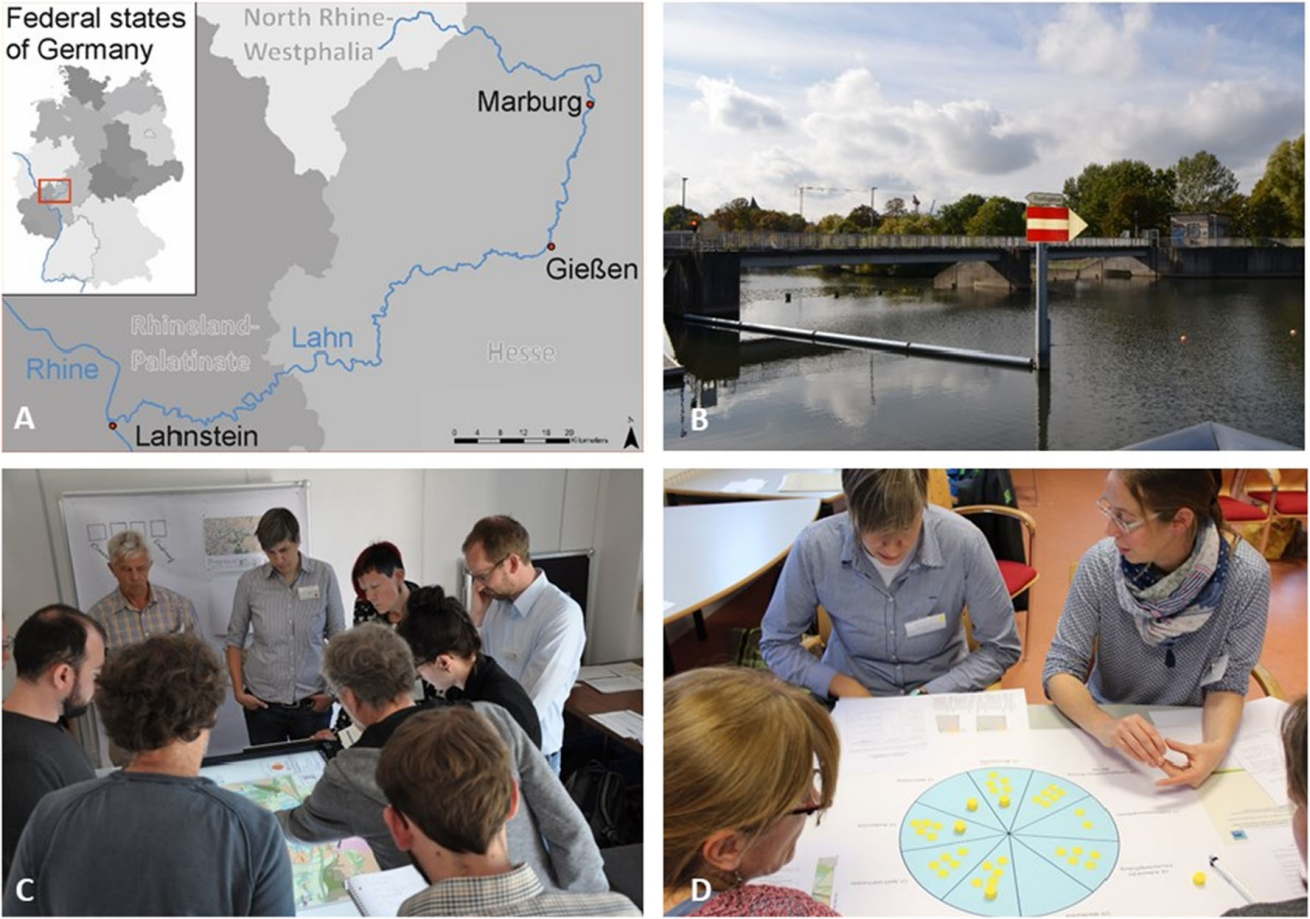
Location of the Lahn River landscape (**a**), Lahn River and technical infrastructure (**b**), and impressions from the LahnLab Workshop Series, depicting a Geodesign-workshop using a touch table and spatial decision support tools (**c**) and a weighing task as part of a participatory multi-criteria analysis (**d**)

Our substantiation of the conceptual framework and reflection on its application draws on insights gained from the transdisciplinary case study of the PlanSmart research project, in which novel approaches to the planning and governance of NBS in river landscapes were investigated (Albert et al. 2019). It focuses on the Lahn River, a tributary to the Rhine, located in Hesse and Rhineland-Palatinate, Germany (Fig. 2a, b). The Lahn was considerably transformed, impacting its linear patency, water regime, and hydrological functionality of parts of its floodplains. In addition, discrepancies still exist with the EU Water Framework Directive’s goals to improve the river’s ecological quality. Societal challenges that could eventually be addressed through NBS include attaining good ecological status according to the Water Framework Directive while mitigating and adapting to climate change impacts such as increased heat stress and flood risks as well as accounting for diverse stakeholder interests including agriculture, hydropower generation, recreational boating and nature conservation.

With the aim of exploring approaches to plan and govern NBS, PlanSmart cooperates with the integrated EU Life Project ‘Living Lahn, one river, many interests’ (LiLa). The LiLa project is a 10-year effort that aims to enhance the river’s ecological health and connectivity according to the standards of the Water Framework Directive while simultaneously enriching humans’ quality of life along its shores (www.lila-livinglahn.de/en/the-project/project-goals). More specifically, this research established a LahnLab as a transdisciplinary platform for cooperation with LiLa Consortium Members and organized a series of workshops (Albert et al. 2019; Fig. 2c) that relate to several of the planning steps outlined in the conceptual framework. The LiLa Consortium also contributed insights from their comprehensive engagement of community stakeholders. The two latter steps of the framework, *Develop solution strategies* and *Realize and monitor*, could not be tested in the scope of the project. An overview of the LahnLab workshops, their respective aims, the number of participants, and relationships to our framework’s planning steps is illustrated in Table 1. The participants of the LahnLab Workshops were representatives of the LiLa Project Consortium of relevant stakeholders from regional (county), state (Hesse and Rhineland-Palatinate), and national level.

**Table 1:** LahnLab Workshop Series conducted in the case study

LahnLab Workshop number and topic	Aim	Relation to planning step (-s)	Number of participants
Workshop 1: Stakeholder interests	Decision upon the objectives for the collaboration between LiLa and [Research Project name omitted in this version] in the LahnLabs. Identification of relevant stakeholders and their respective interests. Definition of a region for investigating the NBS scenarios	– Step 1: Co-define setting – Step 2: Understand challenges	10
Workshop 2: Scenarios	Development of strategic NBS scenarios (‘What if?’) including the assessment of their impact on humans and nature	– Step 3: Create visions and scenarios	11
Workshop 3: Geodesign	Translation of scenario narratives into spatial scenario design of NBS measures using digital Geodesign tools	– Step 3: Create visions and scenarios – Step 4: Assess potential impacts	9
Workshop 4: Multi-criteria analysis	Discussion of NBS options and possibilities for action, i.e. which variants are available for dealing with barrages and what effects the different variants have	– Step 4: Assess potential impacts	11
Workshop 5: Opportunity spaces	Identification of potential areas for the implementation of NBS and evaluation of the method for identification	– Step 3: Create visions and scenarios	7

### Steps of planning nature-based solutions and methods applied in the case study

Over the LahnLab Workshop Series, a range of methods were developed, applied and evaluated for implementing the planning steps in consideration of the procedural planning principles and the NBS criteria. The financial and time investments varied between the steps applied, with most of them requiring expenditures for three to nine months of preparation by a research team of usually three to five members. The degree of consideration of the planning principles and NBS criteria in each method varies (Table 2) and is discussed in the following section. The Supplementary Material (Table S1) provides details on the methods applied

**Table 2:**
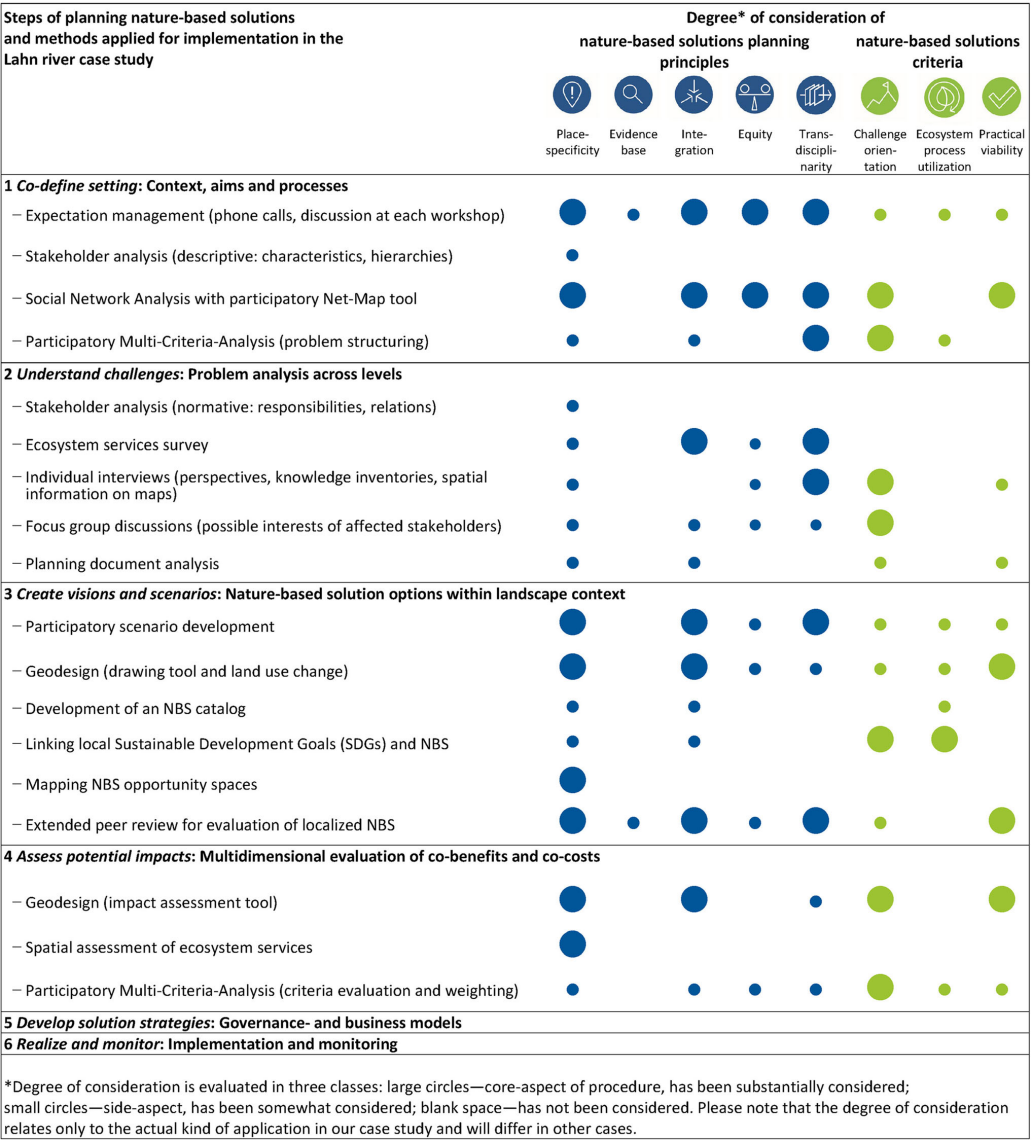
Methods applied in the Lahn River case study to implement the planning steps, and relative degree to which the principles of NBS planning and the criteria for NBS were addressed in each step. For more detailed explanation on each of the methods proposed, please see text and Table S1 in the Supplementary Material

The co-definition of the setting (step 1) was initiated by PlanSmart’s lead team in exchange with practice partners early on during the funding acquisition stage and further operationalized at the project start. Objectives for collaboration were discussed and agreed upon in meetings of the LiLa Steering Committee. A stakeholder analysis provided information on institutional characteristics and hierarchies of all project partners. The first LahnLab Workshop established the context, aims, procedures of work and methods to be applied in the collaboration. A Social Network Analysis based on the participatory Net-Map tool helped detecting important actor relations—information that is essential to understand the governance context.

Furthermore, workshop methods were agreed with LiLa representatives ahead of time, and the specific context and rules were discussed at the beginning of each workshop. Identifying interests instead of harmonizing them was emphasized. For example, contents for a multi-criteria analysis (MCA) were developed with the LiLa Steering Committee to tailor the evaluation of river restoration options to practice needs (Fig. 2d).

To *Understand challenges* (step 2), societal problem dimensions were assessed. Individual semi-structured interviews helped understanding stakeholders’ perspectives, problem orientations and knowledge inventories. Map-drawing provided spatial information on areas with high action needs. The information from the interviews built the basis for the main topics in focus group discussions in the first workshop, in which the participants considered interests of affected stakeholders along the river. Furthermore, a qualitative content analysis of discussion papers on Lahn development goals (IKU 2018) helped understanding objectives of stakeholder groups, identifying similarities in topics and connotations, and deriving synergistic goals. Some biophysical problem dimensions were addressed by assessing and mapping selected ecosystem services based on the river ecosystem service index (Podschun et al. 2018). Another dimension of the problem referring to the mutual relationships between humans and nature was considered by an online public participation GIS survey among local citizens. The survey gave insights into sense of place and the use of the river landscape (Verbrugge et al. 2019). The data was used in a Geodesign Workshop to develop value maps and engage discussion (Fig. 2c). An online survey assessed stakeholders’ involvement in collaboration networks for the co-production of ecosystem services. The Social Network Analysis identified network structures and the level of importance that stakeholders attributed to themselves and others in their collaboration networks.

In order to *Create visions and scenarios* (step 3), we compiled a database containing information on NBS and their contributions to address challenges identified in the previous step. We then selected the NBS that addressed most of the challenges and localized potential implementation areas (opportunity spaces) for these NBS by applying different GIS-based approaches. For instance, we used biophysical spatial information, such as soil type and elevation, to create hydromorphological landscape units to delineate opportunity spaces for NBS (Guerrero et al. 2018). Another approach was to use a Geodesign drawing and land use change tool upon which participants translated and enhanced scenario narratives developed in the previous step. Participants allocated (nature) priority areas, specific measures (e.g. fish ladders, access points to river, recreation trails), and changed land uses accordingly (pasture to wetland forest). An extended peer review involved stakeholders in the quality assurance process and helped to estimate the validity, relevance and viability of opportunity spaces for NBS.

For *Assessing the potential impact* (step 4) of visions and scenarios, different approaches were applied. A Geodesign impact assessment tool was applied to evaluate the impact of land use change for four selected ecosystem services (pollination, recreation, climate regulation, food production) and highlighted potential co-benefits and trade-offs between these ecosystem services. A participatory MCA was applied to evaluate the suitability of different river restoration options to deal with a weir in a Lahn River section. Each participant assessed the performance of multiple criteria for each option and weighted the criteria’s importance according to their values. Small groups discussed individual choices to reflect different viewpoints and reach consensus on criteria performance and weighting. In collaboration with partners, the research team currently applies a multidimensional evaluation method based on spatial indicators for river ecosystem services (Podschun et al. 2018) in the Lahn area. By applying the indicators in scenarios with and without NBS implementation, before-and-after comparison can be visualized.

### Degree of consideration of planning principles and nature-based solutions criteria in the case study

The principle of *Place-specificity* was present in all of our planning steps, with a lesser intensity in step 2. That is because we used methods that were less place-based for the problem analysis of stakeholders and institutions across levels. It was not necessary as the region for investigation was clearly delimited. Working in a place-specific way may require planning participants to have local knowledge, a requirement that may yield conflicts with adhering to the idea of the equity principle.

The principle of *Evidence base* was addressed to a lesser extent in our planning process, only somewhat for expectation management in step 1 and for the evaluation of localized NBS in step 3. Since empirical evidence was unavailable in our case, we designed our research to yield ‘best available’ knowledge. This included providing modelling as ‘expected evidence,’ explicitly communicating uncertainties of evaluations and applying extended peer review methods that best resonate with local knowledge.

The principle of *Integration* was applied across all steps of the planning process undertaken in our case study. The principle of *Integration* could be fully addressed with most of the procedures used in steps 3 and 4. Both steps were covered by the LahnLab Workshop, which provided a platform for integrating knowledge from different spatial (local to national) and governmental levels and across sectoral perspectives. Temporal integration was facilitated, among others, through scenario building exercises to explore plausible futures. Integrated by a common aim and research framework, different disciplinary concepts and methods were applied consecutively, and each built upon insights gained in earlier stages of the planning process. Fine-tuning the design of individual planning steps with support from an external consultant helped gradually developing a coherent and integrated research strategy.

Due to the focus of the case study application on the first four steps of the planning cycle, the principle of *Equity* only applied to the procedural character of the planning process, most strongly in steps 1 and 3. For example, the composition of breakout groups in LahnLab Workshops was intentionally balanced in consideration of issues such as power relations, gender, and disciplinary background. The principle *Equity* concerns the involvement of practitioners and is therefore related to the principle of *Transdisciplinarity.* We found that small groups, mixing stakeholders from different institutions, and involving external moderation were particularly useful for fulfilling the principle.

Finally, the principle of *Transdisciplinarity* was most strongly addressed in steps 1 to 3. In step 1, *Co-define setting*—for expectation management, network analysis and problem structuring—cooperation between science and practice partners was closest. Practitioners were also strongly involved in designing a survey on the co-production of ecosystem services, developing participatory scenarios in step 2, and in the extended peer review for evaluation of localized NBS in step 3. In steps 2 and 4, *Transdisciplinarity* was not fully addressed since we maintained a more analytical level for problem analysis and impact assessment and did not fully involve the practitioners in the methodological design. While the LahnLab facilitated direct integration of practical and scientific knowledge, the colloquial knowledge (cf. Klein 2008) was considered only indirectly through analyses of the outcomes of a comprehensive engagement process conducted by LiLa.

The NBS criteria *Challenge-orientation* and *Practical viability* were present throughout the whole planning process. We interpret this in the way that the NBS concept is more strongly related to social aspects than similar concepts, and that these social aspects have to be addressed throughout the planning process. The criterion of *Ecosystem process utilization* was most intensively considered in steps 3 and 4. We mainly addressed these criteria in the LahnLab Workshops and less so in the problem analysis (step 2), as they are related to the principle of *Place-specificity.*

## Discussion and conclusion

This paper suggested a framework for planning NBS and reflected upon its application in a case study for river management in Germany. The proposed framework resonates with, but also differs from established landscape planning models. Similarities exist in that our framework follows the conventional planning cycle of assessing, goal-setting, designing, implementing and evaluating (cf. Steinitz 1990; von Haaren et al. 2019). However, our framework is innovative in (i) strongly emphasizing an orientation towards specific societal challenges instead of ‘only’ delivering comprehensive plans, (ii) explicitly considering NBS opportunities in the portfolio of potential interventions, (iii) implementing efforts for multidimensional evaluation of planning options that take equity into account, (iv) developing actionable solution strategies as an inherent component of the planning process, and (v) systematically considering governance aspects (in terms of planning principles) and facilitating transdisciplinarity throughout. Generally, implementing the framework will require even more interdisciplinary collaboration within the planning team, including not only planners and ecologists but also social scientists to better understand the interactions between the coupled human–environment systems and to incorporate this knowledge in plan-making.

In comparison with standard landscape planning approaches, applying our framework for planning NBS implies additional complexities. For instance, and in spite of respective expectations (e.g. Nesshöver et al. 2017), we did not find the NBS concept to be easily applicable as a boundary object for stakeholders, some of whom interpreted it as too biased towards nature conservation. We also identified disconnects between the scientific NBS discourse, where definitive clarity is needed, and the debate in practice, where real-world problems need to be addressed regardless of the applied concepts (cf. Hanson et al. 2020). As the NBS concept is little known among German planning practitioners (Brillinger et al. 2020), we adopted only its core ideas but did not engage in lengthy conceptual discourse. Considering the generation of co-benefits as desired impact instead of defining criterion for NBS distinguishes NBS more strongly from similar concepts such as green infrastructure. Extending the consideration of *Equity* beyond the explicit design and execution of the workshop procedures to a bottom-up public involvement would have been advisable but was impossible in the scope of the project. Furthermore, implementing multidimensional evaluation, establishing the knowledge base for *evidence-based* planning and executing *transdisciplinarity* required substantial additional personnel, funds, and time. Our case study also reemphasized that implementing the planning steps rarely happens in a systematic order but rather iteratively, especially in dynamic river landscapes (cf. Grose 2014; Moosavi 2018). For example, scenario exercises helped identifying future challenges, thus complementing problems identified in earlier stages of the planning process.

Given the paper’s focus on suggestion and reflection, the validity and transferability of our results are difficult to evaluate. The framework was developed by considering best available knowledge in the scoping review. The search query applied may have missed publications, and the evaluation of the literature required considerable interpretation, since understandings of NBS and planning processes differ. While our findings from the framework’s application in the case study are supported by documented workshop results, our own reflection may be biased, as are all perceptions.

**Fig. 3:**
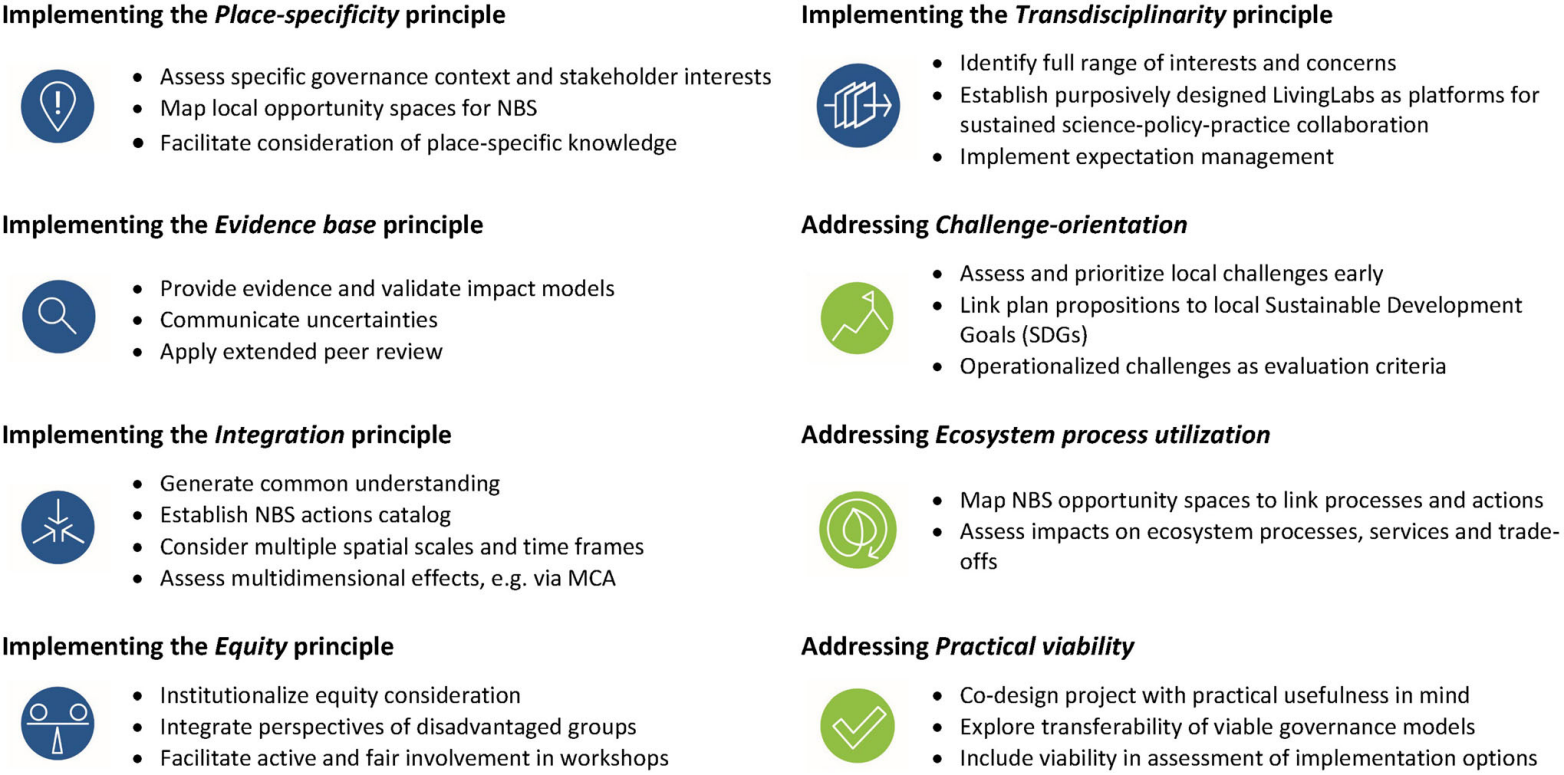
Inspirational checklist of procedures supportive to implement planning principles and address nature-based solutions criteria

Practical application of our framework is advisable on the landscape scale that allows considering larger ecological processes and interactions and resonates with people’s perceptions (Selman 2006). While implementation will often rely on informal planning instruments, coordination with formal instruments such as water management plans is needed to enhance the likelihood of uptake in actual decision-making. To facilitate practical application of our framework, we suggest a checklist of supportive procedures (Fig. 3) that may inspire both the design and execution of planning processes for NBS.

A few issues stand out for further research and experimentation. Clearly, the applicability and adaptability of our framework to different case studies needs to be tested further. Insights gained on additional methods and respective impacts need to be collected and systematically analyzed, especially for the steps of *Develop solution strategies* and *Realize and monitor* which could not be tested in this study. To enhance the evidence base of NBS effectiveness, planning processes need to be designed in consideration of suitable indicators and monitoring systems to evaluate the effects of interventions against pre-defined targets. Finally, research is needed on how to communicate the concept of NBS to stakeholders and which implications the use of the concept may have in stakeholder discussions.

In this paper, we proposed a framework of planning NBS and reflected on its application in a case study. The set of specific planning steps, methods, principles, and NBS criteria included in our framework provide an adaptable approach for NBS planning across multiple contexts. We encourage the practical application of our framework, taking into account the suggested procedures. We also suggest future efforts of planning NBS to consider the following questions: How should the concept of NBS be defined and communicated in the particular context? Which steps of the framework shall be implemented, and with what methods? Which procedures shall be applied to best address the principles? With the framework and the insights shared above, we hope to inspire and guide future research and application, and to contribute to an enhanced consideration of NBS in practice.

## Electronic supplementary material

Below is the link to the electronic supplementary material.

Supplementary file1 (PDF 819 kb)
