# Supplementary material for: Planning nature-based solutions: Principles, steps, and insights
Source: Ambio. 2020 Oct 14;50(8):1446–61. doi: 10.1007/s13280-020-01365-1 (PMC8249551; doi:10.1007/s13280-020-01365-1)
Supplement: Supplementary file 1 — Supplementary file1 (PDF 819 kb) [file 13280_2020_1365_MOESM1_ESM.pdf]

Ambio

Electronic Supplementary Material
This supplementary material has not been peer reviewed

Title: **Planning Nature-based Solutions: Principles, Steps, and Insights**

Christian Albert, Mario Brillinger, Paulina Guerrero, Sarah Gottwald, Jennifer Henze, Stefan Schmidt,

Edward Ott, Barbara Schröter

Table S1: Short description of methods used in the Lahn river landscape case study

Method	Brief description and references
Expectation management	Expectation management was practiced throughout the collaboration in terms of bilateral conversations between the project coordinators and workshop participants and scientists. For instance, participants were explicitly asked about their expectations before the workshop to identify interests and to clarify possible misunderstandings. Furthermore, a brief survey was conducted at the end of each workshop to learn from experiences made and to improve the design and execution of following workshops.
Stakeholder analysis	Aim of a stakeholder analysis is to learn, which stakeholders are the most relevant for the project's purpose. Descriptive approaches like the ones we used have a phenomenological orientation and look at the stakeholders and their interrelations. Characteristics of stakeholders were identified and described and resulting information could be used as input for normative and instrumental approaches (Kimmich et al. 2012; Reed et al. 2008).
Individual interviews	Individual semi-structured interviews were conducted to obtain information about individual perspectives like aims, interests or intentions. For a better comparability we used an interview guideline that contained different thematic blocks with related questions. These can be derived from a theory or lead to the generation of a theory. The analysis of the empirical material gained in the interviews included a two-step coding, following the analytical methods of theoretical coding by Strauss and Corbin (1990).
Social network analysis (SNA) with participatory Net-Map tool	The Net-Map tool is a tool for mapping actors' networks. It detects the important actors in a network and distinctive relations between them by applying four interview steps: 1) recording the influencing actors, 2) identifying links between actors, 3) identifying actors' interests/motivations, and 4) assessing the actors' influence on the network. The data gathered can be used for social network analysis (Hauck and Schiffer 2012).The method has been applied to characterize the network of the LiLa-project partners.
Online public participation geographic information system (PPGIS) survey	PPGIS methods are able to elucidate spatial (and non-spatial) information of local citizens or non-experts to understand the social and cultural landscapes (Brown and Kyttä 2014). In our case study respondents located activities and meaningful places in and around the Lahn river. This information (and thus the voice of the local citizens) was used to support planners or decision makers (Raymond et al. 2014; Gottwald et al. 2020).
Focus group discussion	Focus group discussions in combination with participant observations are used for bringing relevant actors together in order to discuss different perspectives, learn from each other and for the researcher to identify

	group dynamics and interrelations in a more natural conversation framing. Participants can learn from each other through the exchange of experiences, while scientists can provide results relatively quick by talking to several people at once about a certain topic. The method produces data and insights that would be less accessible without interaction found in a group setting that stimulates experiences, ideas and memories by listening to others (Lindlof and Taylor 2002).
Ecosystem services survey	An online survey was conducted amongst local stakeholders to learn about their involvement and the involvement of others on collaboration networks for the co-production of twelve ecosystem services. The data was analyzed with SNA to construct the networks and understand some differences between opposite perspectives. SNA examines social structures using networks and graph theory. Network structures are calculated and visualized according to nodes (actors) and the ties, edges, or links (relationships or interactions) that connect them (Prell 2011).
Development of a nature-based solutions (NBS) catalog	A database was developed containing information on potential NBS for river landscapes (Schmidt et al. in prep). For each of the 646 NBS information was collected on the definition of the NBS, application area, ecological impact, and associated changes in ecosystems services. Also, NBS were assigned to the standardized classification system for generic measures to improve conditions of river landscapes used by the German Working Group on water issues of the Federal States and the Federal Government (LAWA) for the development of action programs and subsequent reporting to the European Commission's Water Information System Europe (WISE) (LAWA 2015). The database content on NBS was compiled through a scoping review (Grant et al. 2009, Tricco et al. 2018) in Web of Science, and grey literature.
Linking local Sustainable Development Goals (SDG) and NBS	For the identification of NBS that contribute to achieve local SDG a qualitative content analysis (Stemler 2001) of development goals in the Lahn river landscape (Best et al. 2019) and the United Nations SDGs (United Nations General Assembly 2015) was conducted. Based on this analysis content-related similarities and differences of development goals were identified (Schmidt et al. in prep). Similar Lahn development goals and SDGs were then linked with NBS according to NBS's major ecological impact and resulting contribution to achieve the development goals. For this step the NBS catalog (see above) was used as well as the classification system developed by Fribourg-Blanc et al. (2015) that determines potential ecological changes due to NBS.
Mapping NBS opportunity spaces	Inspired by hydrological models, which work with delineated hydrological response units, this method is based on hydromorphological landscape units (HLU) which are comprised of specific biophysical spatial criteria to identify potential areas that could function as NBS (Kalcic, Chaubey and Frankenberger 2015; University of Texas A&M 2012). The HLU approach was tested for selected floodplain-related NBS in the Lahn river landscape (Guerrero et al. 2018). Results showed the location and spatial extent of both existing NBS areas and potential areas for NBS

	implementation. The HLU can be used as an initial approach to support NBS planning and implementation.
Participatory scenario development	Implementing NBS at the landscape scale would be a long-term and collaborative effort. Planning with NBS can thus be usefully supported by participatory scenario development. The PlanSmart project applied an exploratory scenario exercise in which the future context conditions were envisioned as a combination of two key drivers: the level of consideration of ecosystem processes in river management, and the governance context (ranging from entirely market-driven to more state-controlled). The combination of those two drivers opened up four quadrants which were subsequently jointly developed into storylines and hand-drawn visualizations of how the future landscape might look like. Participants were further asked to choose between a handful of roles (in terms of characters such as a farmer or journalist) and were exposed to a time travel-exercise to support an engagement with potential futures and an open-minded exploration of potential and plausible future scenarios.
Geodesign	Geodesign can be defined as 'a design and planning method, which tightly couples the creation of design proposals with impact simulations informed by geographic contexts, systems thinking and digital technology' (Steinitz 2012, p. 12). Geodesign provides useful tools and methods to facilitate participatory planning, manage boundaries between participants, and assess impacts of NBS. The Geodesign process was implemented in PlanSmart as a workshop consisting of five steps to co-develop and explore spatial scenario maps in consideration of NBS. The steps included (i) a recap of scenario stories developed in a prior workshop, (ii) scenario sketching, (iii) allocating land uses, (iv) exploring impacts, and (v) reflecting.
Extended peer review for evaluation of localized NBS	Extended peer review is the involvement of non-academic actors in the quality assurance process of knowledge generation (Funtowicz et al. 2015). By using additional knowledge from stakeholders localized NBS can be evaluated and scenarios for landscape planning prioritized. In PlanSmart an extended peer review was conducted in a one-day workshop involving nine representatives from local to national, governmental authorities responsible for Lahn development strategies and actions (Schmidt et al. in prep). The workshop was structured as follows: i) Briefing on topic and tasks through informative presentation of scientific findings (localized NBS) and introduction of quality assurance tasks; ii) evaluation of the quality of the scientific work and documentation of explicit value judgements by peers through open round table discussions and interactive GIS-based approaches; iii) reflection of results through discussions and revision of evaluation results.
Governance model database analysis	Although co-designing governance or business models for implementation was not possible in the scope of the case study, a database of successful NBS projects on similar issues within Germany was developed. The database was used to identify determinants of success and clusters of combinations of variables that together provide

	inspiration for designing place-specific governance models (Zingraff-Hamed et al. in review).
Planning document analysis	Planning documents can be considered outcomes of decision-making processes. They contain information on the discourse of specific challenges, measures, planning processes and decision practices at a certain point in time. A document analysis aims to capture and analyze the contents of planning documents to reconstruct past planning processes, and adapts methods of qualitative and quantitative content analysis (Brillinger et al. 2020).
Multi-criteria analysis (MCA)	MCA is an umbrella term to describe a set of approaches that can be applied to structure and inform decision-making. The basic idea of MCA methods is to evaluate the performances of alternatives (e.g. management options) with respect to criteria that capture the key dimensions of the challenge (e.g. ecological, social, and economical criteria), involving human judgments and preferences. MCA commonly include (i) problem structuring that defines alternatives and evaluation criteria, (ii) criteria evaluation that assess the performance of each alternative with respect to multiple criteria (scoring) and attaching the importance to the criteria (weighting), (iii) application of an aggregation rule that combine criteria scores and weights to derive overall values of each alternative, and (iv) actual decision on the suitability of alternatives (Adem Esmail and Geneletti 2018). The combination of an MCA with participatory and deliberative elements is useful in dealing with the plurality of values and beliefs held by stakeholders on certain types of challenges. MCA should be as participative and transparent as possible to increase the relevance for the stakeholders and legitimacy of the evaluation process (Greco and Munda, 2017).
Spatial assessment of ecosystem services, also as co-benefits of NBS	Spatial assessment of ecosystem services is a geographic analysis and valuation which seeks to explain patterns of nature's contribution to human well-being and its spatial evidence. In collaboration with PlanSmart the River Ecosystem Service Index (RESI) was applied for spatial assessment of ecosystem services in the Lahn river landscape. The RESI is an assessment approach for quantitative valuation and spatial explicit mapping of 16 ecosystem services of river landscapes including provisioning (e.g. crops, plant biomass), regulating (e.g. flood regulation, nitrogen retention) and cultural ecosystem services (e.g. landscape aesthetic, water-related activities) (Podschun et al. 2018). In the RESI ecosystem services are defined by spatial indicators that represent major socio-ecological conditions of the river landscape. For each of the indicators spatial data and a GIS for data processing is required. Outputs of RESI are standardized into a five-tier scale to enable integrative valuation and summary across different river segments. By assessing the RESI before and after the implementation of NBS, or scenarios of different NBS, the impact on ES can be estimated and planning alternatives discussed (Podschun et al.2018).

References used in Table S1

Best, R., K. Geisler, H. J. Grommelt, D. Heinz, D. Jürgens, M. Korn, T. Norgall, M. Sommerhage, and A. Zedler. 2019. Erlebnisreiche Lebensader Lahn. Anregungen zum Lahnkonzept LiLa-Living Lahn 2025, NABU Landesverband Hessen, BUND, HGON, (pp. 1-75).

Brillinger, M., A. Dehnhardt, R. Schwarze, C. Albert. 2020. Exploring the uptake of nature-based measures in flood risk management: Evidence from German federal states. Environ. Sci. Policy 110, 14–23. https://doi.org/10.1016/j.envsci.2020.05.008Brown, G., and M. Kyttä. 2014. Key issues and research priorities for public participation GIS (PPGIS): A synthesis based on empirical research. *Applied Geography* 46: 122–136.

Eggermont, H., E. Balian, J. M. N. Azevedo, V. Beumer, T. Brodin, J. Claudet, B. Fady, M. Grube et al. 2015. Nature-based Solutions: New Influence for Environmental Management and Research in Europe. *GAIA - Ecological Perspectives for Science and Society* 24: 243–248.

Adem Esmail, B., and D. Geneletti. 2018. Multi-criteria decision analysis for nature conservation: A review of 20 years of applications. Methods Ecol. Evol. 9, 42–53. https://doi.org/10.1111/2041-210X.12899

European Commission. 2015. Towards an EU Research and Innovation policy agenda for Nature-Based Solutions & Re-Naturing Cities (Final Report of the Horizon 2020 Expert Group on Nature-Based Solutions and Re-Naturing Cities). Brussels, Belgium.

Funtowicz, S.O. and J. R. Ravetz. 2015. Peer Review and Quality Control. In: *International Encyclopedia of the Social & Behavioral Sciences (Second Edition),* ed. J. D. Wright, 680–684. Oxford, Springer.

Gottwald, S., R. Janssen, and C. M. Raymond. 2020. Can Geodesign be used to facilitate boundary management for planning and implementation of Nature Based Solutions? In: Modelling Nature-based Solutions: Integrating Computational and Participatory Scenario Modelling for Environmental Management and Planning, ed. N. Sang, Cambridge: Cambridge University Press.

Grant, M. J., and A. Booth. 2018. A typology of reviews: an analysis of 14 review types and associated methodologies. *Health information and libraries journal*, 26(2): 91–108.

Greco, S., Munda, G., 2018. Multiple Criteria Evaluation in Environmental Policy Analysis. *Routledge Handbook of Ecological Economics.* Routledge Handbooks Online, 311–320.

Guerrero, P., D. Haase, and C. Albert. 2018. Locating Spatial Opportunities for Nature-Based Solutions: A River Landscape Application. *Water* 10: 1–15.

Hauck, J., and E. Schiffer. 2012. Between Intuition and Indicators. Using Net-Map for Visual and Qualitative Social Network Analysis. In: Knoten und Kanten 2.0: Soziale Netzwerkanalyse in Medienforschung und Kulturanthropologie, ed. M. Gamper. 231–257. Bielefeld. transcript.

Kalcic, M., I. Chaubey, and J. Frankenberger. 2015. Defining Soil and Water Assessment Tool (SWAT) Hydrologic Response Units (HRUs) by Field Boundaries. *International Journal of Agricultural and Biological Engineering* 8 (3): 69–80.

Nesshöver C., T. Assmuth, K. N. Irvine, G. M. Rusch, K. A. Waylen, B. Delbaere, D. Haase, and L. Jones-Walters et al. 2017. The science, policy and practice of nature-based solutions: An interdisciplinary perspective. *Science of the Total Environment* 579: 1115 – 1127.

Podschun, S. A., C. Albert, G. Costea, C. Damm, A. Dehnhardt, C. Fischer, H. Fischer, H. Foeckler, et al. 2018. RESI – Anwendungshandbuch: Ökosystemleistungen von Flüssen und Auen erfassen und bewerten. IGB, IGB-Berichte Heft 31/2018, Berlin.

Prell, C. 2011. Social Network Analysis: History, Theory and Methodology, Sage Publications, Los Angeles, London, New Delhi, Singapore, and Washington, D.C, pp. 272

Raymond, C. M., J. O. Kenter, T. Plieninger, N. J. Turner, and K. A. Alexander. 2014. Comparing instrumental and deliberative paradigms underpinning the assessment of social values for cultural ecosystem services. *Ecological Economics* 107: 145–156.

Schmidt, S., C. Albert, and P. Guerrero. (in prep). Nature-based solutions in the Lahn River landscape, Germany – A contribution to localize Sustainable Development Goals.

Steinitz, C. 2012. A framework for geodesign: changing geography by design. Esri.

Stemler, S.E. 2015. Content Analysis. *Emerging Trends in the Social and Behavioral Sciences* 7 (17): 1-14.

Strauss, A. and Corbin, J. 1990. *Basics of Qualitative Research.* Newbury Park, CA: Sage.

Tricco, A. C., W. Zarin, M. Ghassemi, V. Nincic, E. Lillie, M. J. Page, L. Shamseer, J. Antony, P. Rios, J. Hwee, A. A. Veroniki, D. Moher, L. Hartling, B. Pham, and S. E. Straus. 2018. Same family, different species: methodological conduct and quality varies according to purpose for five types of knowledge synthesis. *Journal of Clinical Epidemiology* 96:133-142.United Nations General Assembly. 2015. A/RES/70/1 - Transforming our world: The 2030 Agenda for Sustainable Development.

University of Texas A & M. 2012. *SWAT Input/Ouput File Documentation. Soil and Water Assessment Tool*.

BOX S1: Information on scoping review

The scoping review and synthesis of the best available knowledge regarding the framework of planning NBS considered all relevant literature that could be found by a dedicated query in , performed on November 6, 2019. In our search query title, abstracts and keywords of publication were screened for the following search terms: 'principle* OR criteria OR criterion OR characteristic* OR implication* OR strategy* OR guideline* OR guide* OR standard* OR basis OR rule* OR require* OR law* OR framework* AND "nature-based solution*" OR "nature based solution*'. We identified 238 publications and discarded 179 after thoroughly reviewing titles and abstracts. Studies that used NBS only as buzzword without detailed information on NBS or NBS planning were excluded. For the remaining 59 publications, full text reviews were conducted. Additionally, two frequently cited policy reports (European Commission 2015; and Cohen-Shacham et al. 2016) were included.

Table S2: Literature included in synthesis and refinement of the framework for planning NBS.

**Literature**
Albert C., Spangenberg J.H., Schröter B., 2017, Nature-based solutions: Criteria, *Nature*, 543 (7645), 315, doi: 10.1038/543315b
Albert, C., Schröter B., Haase D., Brillinger M., Henze J., Herrmann S., Gottwald S., Guerrero P., Nicolas C., Matzdorf B., 2019, Addressing societal challenges through nature-based solutions: How can landscape planning and governance research contribute?, *Landscape and Urban Planning*, 182, 12-21, doi: 10.1016/j.landurbplan.2018.10.003
Arkema K.K., Griffin R., Maldonado S., Silver J., Suckale J., Guerry A.D., 2017, Linking social, ecological, and physical science to advance natural and nature-based protection for coastal communities, *Annals of the New York Academy of Sciences*, 1399 (1), 5-26, doi: 10.1111/nyas.13322
Badiu D.L., Nita A., Iojă C.I., Niţă M.R., 2019, Disentangling the connections: A network analysis of approaches to urban green infrastructure, *Urban Forestry and Urban Greening*, 41, 211-220, doi: 10.1016/j.ufug.2019.04.013
Bokhove O., Kelmanson M.A., Kent T., Piton G., Tacnet J.-M., 2019, Communicating (nature-based) flood-mitigation schemes using flood-excess volume, *River Research and Applications*, doi: 10.1002/rra.3507
Bridgewater P., 2018, Whose nature? What solutions? Linking Ecohydrology to Nature-based solutions, *Ecohydrology and Hydrobiology*, 18 (4), 311-316, doi: 10.1016/j.ecohyd.2018.11.006
Bush J., Doyon A., 2019, Building urban resilience with nature-based solutions: How can urban planning contribute?, *Cities*, 95. doi: 10.1016/j.cities.2019.102483
Calliari E., Staccione A., Mysiak J., 2019, An assessment framework for climate-proof nature-based solutions, *Science of the Total Environment*, 656, 691-700, doi: 10.1016/j.scitotenv.2018.11.341
Cilliers E.J., 2019, Reflecting on green infrastructure and spatial planning in Africa: The complexities, perceptions, and way forward, *Sustainability*, 11 (2), doi: 10.3390/su11020455
Cohen-Shacham E., Andrade A., Dalton J., Dudley N., Jones M., Kumar C., Maginnis S., Maynard S., Nelson C.R., Renaud F.G., Welling R., Walters G., 2019, Core principles for successfully implementing and upscaling Nature-based Solutions, *Environmental Science and Policy*, 98, 20-29, doi: 10.1016/j.envsci.2019.04.014
Colléony A., Shwartz A., 2019, Beyond assuming co-benefits in nature-based solutions: A human-centered approach to optimize social and ecological outcomes for advancing sustainable urban planning, *Sustainability*, 11 (18), doi: 10.3390/su11184924
Connop S., Vandergert P., Eisenberg B., Collier M.J., Nash C., Clough J., Newport D., 2015, Renaturing cities using a regionally-focused biodiversity-led multifunctional benefits approach to urban green infrastructure, *Environmental Science and Policy*, 62, 99-111, doi: 10.1016/j.envsci.2016.01.013
Davies C., Lafortezza R., 2019, Transitional path to the adoption of nature-based solutions, *Land Use Policy*, 80, 406-409, doi: 10.1016/j.landusepol.2018.09.020
Dorst H., van der Jagt A., Raven R., Runhaar H., 2019, Urban greening through nature-based solutions – Key characteristics of an emerging concept, *Sustainable Cities and Society*, 49, doi: 10.1016/j.scs.2019.101620

Egusquiza A., Cortese M., Perfido D., 2019, Mapping of innovative governance models to overcome barriers for nature based urban regeneration, *IOP Conference Series: Earth and Environmental Science*, 323 (1), doi: 10.1088/1755-1315/323/1/012081

Faivre N., Fritz M., Freitas T., de Boissezon B., Vandewoestijne S., 2017, Nature-Based Solutions in the EU: Innovating with nature to address social, economic and environmental challenges, *Environmental Research*, 159, 509-518, doi: 10.1016/j.envres.2017.08.032

Fernandes J.P., Guiomar N., 2018, Nature-based solutions: The need to increase the knowledge on their potentialities and limits, *Land Degradation and Development*, 29 (6), 1925-1939, doi: 10.1002/ldr.2935

Fernandes J.P., Guiomar N., Gil A., 2019, Identifying key factors, actors and relevant scales in landscape and conservation planning, management and decision making: Promoting effective citizen involvement, *Journal for Nature Conservation*, 47, 12-27, doi: 10.1016/j.jnc.2018.11.001

Fink H.S., 2016, Human-nature for climate action: Nature-based solutions for urban sustainability, *Sustainability*, 8 (3), doi: 10.3390/su8030254

Frantzeskaki N., 2019, Seven lessons for planning nature-based solutions in cities, *Environmental Science and Policy*, 93, 101-111, doi: 10.1016/j.envsci.2018.12.033

Gómez Martín E., Máñez Costa M., Schwerdtner Máñez K., 2020, An operationalized classification of Nature Based Solutions for water-related hazards: From theory to practice, *Ecological Economics*, 167, doi: 10.1016/j.ecolecon.2019.106460

Gopalakrishnan V., Bakshi B.R., 2017, Including Nature in Engineering Decisions for Sustainability, *Encyclopedia of Sustainable Technologies*, 107-116, doi: 10.1016/B978-0-12-409548-9.10039-9

Guerrero P., Haase D., Albert C., 2018, Locating spatial opportunities for nature-based solutions: A river landscape application, *Water*, 10 (12), doi: 10.3390/w10121869

Gulsrud N.M., Hertzog K., Shears I., 2018, Innovative urban forestry governance in Melbourne?: Investigating “green placemaking” as a nature-based solution, *Environmental Research*, 161, 158-167, doi: 10.1016/j.envres.2017.11.005

Hernández-Morcillo M., Burgess P., Mirck J., Pantera A., Plieninger T., 2018, Scanning agroforestry-based solutions for climate change mitigation and adaptation in Europe, *Environmental Science and Policy*, 80, 44-52, doi: 10.1016/j.envsci.2017.11.013

Izydorczyk K., Piniewski M., Krauze K., Courseau L., Czyż P., Giełczewski M., Kardel I., Marcinkowski P., Szuwart M., Zalewski M., Frątczak W., 2019, The ecohydrological approach, SWAT modelling, and multi-stakeholder engagement – A system solution to diffuse pollution in the Pilica basin, Poland, *Journal of Environmental Management*, 248, doi: 10.1016/j.jenvman.2019.109329

La Notte A., 2018, Accounting for the ecosystem services generated by Nature-based Solutions to measure urban resilience. A methodological proposal, *Economics and Policy of Energy and the Environment*, 2018 (2), 43-61, doi: 10.3280/EFE2018-002003

Lafortezza R., Chen J., van den Bosch C.K., Randrup T.B., 2018, Nature-based solutions for resilient landscapes and cities, *Environmental Research*, 165, 431-441, doi: 10.1016/j.envres.2017.11.038

Lafortezza R., Sanesi G., 2019, Nature-based solutions: Settling the issue of sustainable urbanization, *Environmental Research*, 172, 394-398, doi: 10.1016/j.envres.2018.12.063

Liski A.H., Ambros P., Metzger M.J., Nicholas K.A., Wilson A.M.W., Krause T., 2019, Governance and stakeholder perspectives of managed re-alignment: adapting to sea level rise in the Inner Forth estuary, Scotland, *Regional Environmental Change*, doi: 10.1007/s10113-019-01505-8

Loiseau E., Saikku L., Antikainen R., Droste N., Hansjürgens B., Pitkänen K., Leskinen P., Kuikman P., Thomsen M., 2016, Green economy and related concepts: An overview, *Journal of Cleaner Production*, 139, 361-371, doi: 10.1016/j.jclepro.2016.08.024

Mabon L., 2019, Enhancing post-disaster resilience by ‘building back greener’: Evaluating the contribution of nature-based solutions to recovery planning in Futaba County, Fukushima Prefecture, Japan, *Landscape and Urban Planning*, 187, 105-118, doi: 10.1016/j.landurbplan.2019.03.013

Maes J., Jacobs S., 2017, Nature-Based Solutions for Europe's Sustainable Development, *Conservation Letters*, 10 (1), 121-124, doi: 10.1111/conl.12216

Moosavi S., 2017, Ecological Coastal Protection: Pathways to Living Shorelines, *Procedia Engineering*, 196, 930-938, doi: 10.1016/j.proeng.2017.08.027

Morris R., Strain E.M.A., Konlechner T.M., Fest B.J., Kennedy D.M., Arndt S.K., Swearer S.E., 2019, Developing a nature-based coastal defence strategy for Australia, *Australian Journal of Civil Engineering*, doi: 10.1080/14488353.2019.1661062

Narayan S., Reguero B.G., Van Wesenbeeck B., Burkes-Copes K.A., Losada I.J., Beck M.W., Ingram J.C., 2015, Bridging the Gap between Engineering and Ecology: Towards a Common Framework for Conventional and Nature-Based Coastal Defenses, Coastal Structures and Solutions to Coastal Disasters 2015: Resilient Coastal Communities - *Proceedings of the Coastal Structures and Solutions to Coastal Disasters Joint Conference* 2015, 375-384, doi: 10.1061/9780784480304.040

Nesshöver C., Assmuth T., Irvine K.N., Rusch G.M., Waylen K.A., Delbaere B., Haase D., Jones-Walters L., Keune H., Kovacs E., Krauze K., Külvik M., Rey F., van Dijk J., Vistad O.I., Wilkinson M.E., Wittmer H., 2017, The science,

policy and practice of nature-based solutions: An interdisciplinary perspective, *Science of the Total Environment*, 579 1215-1227, doi: 10.1016/j.scitotenv.2016.11.106

Pagano A., Pluchinotta I., Pengal P., Cokan B., Giordano R., 2019, Engaging stakeholders in the assessment of NBS effectiveness in flood risk reduction: A participatory System Dynamics Model for benefits and co-benefits evaluation, *Science of the Total Environment*, 690, 543-555, doi: 10.1016/j.scitotenv.2019.07.059

Pelorosso R., Gobattoni F., Leone A., 2018, Increasing hydrological resilience employing nature-based solutions: A modelling approach to support spatial planning, *Green Energy and Technology*, PartF12 (), 71-82, doi: 10.1007/978-3-319-77682-8_5

Pontee N., Narayan S., Beck M.W., Hosking A.H., 2016, Nature-based solutions: Lessons from around the world, *Proceedings of the Institution of Civil Engineers: Maritime Engineering*, 169 (1), 29-36, doi: 10.1680/jmaen.15.00027

Raymond C.M., Frantzeskaki N., Kabisch N., Berry P., Breil M., Nita M.R., Geneletti D., Calfapietra C., 2017, A framework for assessing and implementing the co-benefits of nature-based solutions in urban areas, *Environmental Science and Policy*, 77, 15-24, doi: 10.1016/j.envsci.2017.07.008

Rice L., 2019, Nature-based solutions for urban development and tourism, *International Journal of Tourism Cities*, doi: 10.1108/IJTC-05-2019-0069

Santoro S., Pluchinotta I., Pagano A., Pengal P., Cokan B., Giordano R., 2019, Assessing stakeholders' risk perception to promote Nature Based Solutions as flood protection strategies: The case of the Glinščica river (Slovenia), *Science of the Total Environment*, 655 188-201, doi: 10.1016/j.scitotenv.2018.11.116

Sarabi S.E., Han Q., Romme A.G.L., de Vries B., Wendling L., 2019, Key enablers of and barriers to the uptake and implementation of nature-based solutions in urban settings: A review, *Resources*, 8 (3), doi: 10.3390/resources8030121

Schoonees T., Gijón Mancheño A., Scheres B., Bouma T.J., Silva R., Schlurmann T., Schüttrumpf H., 2019, Hard Structures for Coastal Protection, Towards Greener Designs, *Estuaries and Coasts*, doi: 10.1007/s12237-019-00551-z

Scott M., Lennon M., Haase D., Kazmierczak A., Clabby G., Beatley T., 2016, Nature-based solutions for the contemporary city/Re-naturing the city/Reflections on urban landscapes, ecosystems services and nature-based solutions in cities/Multifunctional green infrastructure and climate change adaptation: brownfield greening as an adaptation strategy for vulnerable communities?/Delivering green infrastructure through planning: insights from practice in Fingal, Ireland/Planning for biophilic cities: from theory to practice, *Planning Theory and Practice*, 17 (2), 267-300, doi: 10.1080/14649357.2016.1158907

Short C., Clarke L., Carnelli F., Uttley C., Smith B., 2019, Capturing the multiple benefits associated with nature-based solutions: Lessons from a natural flood management project in the Cotswolds, UK, *Land Degradation and Development*, 30 (3), 241-252, doi: 10.1002/ldr.3205

Song Y., Kirkwood N., Maksimović Č., Zhen X., O'Connor D., Jin Y., Hou D., 2019, Nature based solutions for contaminated land remediation and brownfield redevelopment in cities: A review, *Science of the Total Environment*, 663, 568-579, doi: 10.1016/j.scitotenv.2019.01.347

Turkelboom F., Leone M., Jacobs S., Kelemen E., García-Llorente M., Baró F., Termansen M., Barton D.N., Berry P., Stange E., Thoonen M., Kalóczkai Á., Vadineanu A., Castro A.J., Czúcz B., Röckmann C., Wurbs D., Odee D., Preda E., Gómez-Baggethun E., Rusch G.M., Pastur G.M., Palomo I., Dick J., Casaer J., van Dijk J., Priess J.A., Langemeyer J., Mustajoki J., Kopperoinen L., Baptist M.J., Peri P.L., Mukhopadhyay R., Aszalós R., Roy S.B., Luque S., Rusch V., 2018, When we cannot have it all: Ecosystem services trade-offs in the context of spatial planning, *Ecosystem Services*, 29 (), 566-578, doi: 10.1016/j.ecoser.2017.10.011

van der Jagt A.P.N., Raven R., Dorst H., Runhaar H., 2019, Nature-based innovation systems, *Environmental Innovation and Societal Transitions*. doi: 10.1016/j.eist.2019.09.005

van der Jagt A.P.N., Smith M., Ambrose-Oji B., Konijnendijk C.C., Giannico V., Haase D., Lafortezza R., Nastran M., Pintar M., Železnikar, Cvejić R., 2019, Co-creating urban green infrastructure connecting people and nature: A guiding framework and approach, *Journal of Environmental Management*, 233, 757-767, doi: 10.1016/j.jenvman.2018.09.083

Wamsler C., Niven L., Beery T.H., Bramryd T., Ekelund N., Jönsson K.I., Osmani A., Palo T., Stålhammar S., 2016, Operationalizing ecosystem-based adaptation: Harnessing ecosystem services to buffer communities against climate change, *Ecology and Society*, 21 (1), doi: 10.5751/ES-08266-210131

Wendling L.A., Huovila A., zu Castell-Rüdenhausen M., Hukkalainen M., Airaksinen M., 2018, Benchmarking nature-based solution and smart city assessment schemes against the sustainable development goal indicator framework, *Frontiers in Environmental Science*, 6 (JUL), doi: 10.3389/fenvs.2018.00069

Xing Y., Jones P., Donnison I., 2017, Characterisation of nature-based solutions for the built environment, *Sustainability*, 9 (1), doi: 10.3390/su9010149

Young A.F., Marengo J.A., Martins Coelho J.O., Scofield G.B., de Oliveira Silva C.C., Prieto C.C., 2019, The role of nature-based solutions in disaster risk reduction: The decision maker's perspectives on urban resilience in São Paulo state, *International Journal of Disaster Risk Reduction*, 39, doi: 10.1016/j.ijdrr.2019.101219

Zalewski M., Arduino G., Bidoglio G., Junk W., Cullmann J., Uhlenbrook S., Xia J., de Leaniz C.G., Rowinski P.M., Vörösmarty C.J., Chicharo L., 2018, Low cost, nature-based solutions for managing aquatic resources:

integrating the principles of Ecohydrology and the Circular Economy, *Ecohydrology and Hydrobiology*, 18 (4), 309-310, doi: 10.1016/j.ecohyd.2018.12.001
Zölch T., Wamsler C., Pauleit S., 2018, Integrating the ecosystem-based approach into municipal climate adaptation strategies: The case of Germany, *Journal of Cleaner Production*, 170, 966-977, doi: 10.1016/j.jclepro.2017.09.146
Zuniga-Teran A.A., Staddon C., de Vito L., Gerlak A.K., Ward S., Schoeman Y., Hart A., Booth G., 2019, Challenges of mainstreaming green infrastructure in built environment professions, *Journal of Environmental Planning and Management*, doi: 10.1080/09640568.2019.1605890
Zwierzchowska I., Fagiewicz K., Poniży L., Lupa P., Mizgajski A., 2019, Introducing nature-based solutions into urban policy – facts and gaps. Case study of Poznań, *Land Use Policy*, 85, 161-175, doi: 10.1016/j.landusepol.2019.03.025
